# The emerging roles of interstitial macrophages in pulmonary fibrosis: A perspective from scRNA-seq analyses

**DOI:** 10.3389/fimmu.2022.923235

**Published:** 2022-09-22

**Authors:** Yanrong Gu, Toby Lawrence, Rafeezul Mohamed, Yinming Liang, Badrul Hisham Yahaya

**Affiliations:** ^1^ Laboratory of Genetic Regulators in the Immune System, Henan Collaborative Innovation Center of Molecular Diagnosis and Laboratory Medicine, School of Laboratory Medicine, Xinxiang Medical University, Xinxiang, China; ^2^ Lung Stem Cells and Gene Therapy Group, Department of Biomedical Sciences, Advanced Medical and Dental Institute (AMDI), Universiti Sains Malaysia, Bertam, Kepala Batas, Malaysia; ^3^ Henan Key Laboratory of Immunology and Targeted Therapy, School of Laboratory Medicine, Xinxiang Medical University, Xinxiang, China; ^4^ Centre for Inflammation Biology and Cancer Immunology, Cancer Research UK King’s Health Partners Centre, School of Immunology and Microbial Sciences, King’s College London, London, United Kingdom

**Keywords:** lung macrophage classification, interstitial macrophages, scRNA-seq, heterogeneity of interstitial macrophages, pulmonary fibrosis

## Abstract

Pulmonary fibrosis is an irreversible and progressive disease affecting the lungs, and the etiology remains poorly understood. This disease can be lethal and currently has no specific clinical therapeutic regimen. Macrophages, the most common type of immune cell in the lungs, have been reported to play a key role in the pathogenesis of fibrotic disease. The lung macrophage population is mostly composed of alveolar macrophages and interstitial macrophages, both of which have not been thoroughly studied in the pathogenesis of lung fibrosis. Interstitial macrophages have recently been recognised for their participation in lung fibrosis due to new technology arising from a combination of bioinformatics and single-cell RNA sequencing analysis. This paper reviews recent developments regarding lung macrophage classification and summarizes the origin and replenishment of interstitial macrophages and their function in pulmonary fibrosis.

## Introduction

Pulmonary fibrosis is an irreversible chronic lung disease mainly characterized by increased collagen deposition and collapse of lung structure. Idiopathic pulmonary fibrosis (IPF) is an incurable disease related to age, gender, family, and environmental factors (1). Pulmonary fibrosis is caused by abnormal tissue repair, resulting in widespread scarring of the lungs. In addition, high deposition of extracellular matrix (ECM) and aberrant fibroblast activity have been previously detected in the lungs of IPF patients (2). As a result, patient lung function deteriorates and pulmonary gas exchange is impaired, resulting in clinical manifestations of dry cough, shortness of breath, and significantly decreased quality of life (2, 3). So far, the underlying mechanisms involved in IPF development are unclear despite the incidence of disease increasing over the last several decades (4–6). The average survival time after IPF diagnosis is generally three to five years without effective treatment (7). It is thought to have a worsened prognosis compared to many cancers because of its high lethality (8). Although two drugs, namely Nintedanib and Pirfenidone, are recognised as effective therapies for this disease (9, 10), they only alleviate disease progression and do not address damage that has already occurred. It is generally believed that lung transplantation is the only solution for IPF (3), but less than half of IPF patients live more than 5 years after transplantation (1, 11–13). This shortened lifespan may be attributable to the age of the patient, the condition of the donor lung, the timing of the referral for transplantation, and the surgical approach (11, 12). The underlying mechanisms that affect life span are still worth exploring. Therefore, understanding the pathophysiology of pulmonary fibrosis is a critical aspect of improving patient outcomes.

Since pulmonary fibrosis is also an inflammatory disease (3), it is vital to explore the role of immune cells in disease pathogenesis. Macrophages are the most common immune cell found in the lungs and they play a crucial role in immune responses and airway remodelling during pulmonary fibrosis (14). Most of the chemokines and cytokines found in lung fibrosis are associated with macrophages (15). Due to the heterogeneity and plasticity seen in macrophages from healthy and diseased lungs, it has been difficult to sufficiently study pulmonary macrophages, especially their classification and function during lung fibrosis. With the advent of single-cell RNA sequencing (scRNA-seq), the characterization of macrophage heterogeneity has been improved in murine pulmonary fibrosis models and in patients with lung fibrosis.

This review summarizes the classification of pulmonary macrophages driven by scRNA-seq technology and highlights the role of interstitial macrophages in lung fibrosis. Elucidating the key immunological features involved in lung fibrosis using single-cell multi-omics may help identify new therapeutic targets for clinical treatment.

## The canonical classification of lung macrophages

Macrophages in the lung are generally divided into two classical categories according to their location. Alveolar macrophages (AMs) are usually located in the airway and interstitial space, while interstitial macrophages (IMs) exist in the parenchyma of the lung (16–19). Universal markers for lung macrophages include MERTK, a tyrosine kinase, which is also expressed in microglia, and CD64 encoded by *fcgr1a*, a reliable marker (20–23). In addition, both IMs and AMs express CD64 and MERTK, which distinguishes them from monocytes and conventional dendritic cells (22). More recent attempts in categorizing macrophages based on immunophenotyping are inconsistent, however, the most commonly used markers to differentiate AMs in murine include high autofluorescence, SiglecF, and CD11c, while markers to differentiate IMs are CD11b and CX3CR1 (22, 24–26). However, in humans, there are relatively few surface markers for AMs and IMs as lung parenchyma from healthy samples is extremely difficult to obtain (27). HLA-DR, CD11b, CD169, CD206, and mononuclear markers, such as CD16 and CD14, have been used to effectively identify pulmonary macrophages in humans (27–29). The common AMs and IMs-specific recognition markers used in humans and mice are summarized in **Table 1**. Furthermore, the origin, replenishment, morphology, and function of AMs and IMs are compared in **Table 2**. Classifying lung macrophages by location is useful but may not accurately reflect the exact heterogeneity of macrophages in healthy and diseased lungs. Indeed, in-depth analyses using genetic tools to trace cellular differentiation have revealed that location alone may not be enough to define these two pulmonary macrophage subsets (46). Interestingly, IMs have been found to possess the potential for conversion to AMs during pulmonary fibrosis based on phenotype (30, 46, 47). Therefore, an updated classification strategy for lung macrophages in pulmonary fibrosis is necessary. In addition, more in-depth analyses of lung macrophages can help delineate the apparent heterogeneity and provide opportunities to elucidate more specific features of each lung macrophage subset during fibrosis.

**Table 1:** Identification markers of AM and IM in steady state.

	Shared markers	Identification makers	Reference
In mouse			
AM	F4/80, CD64, Mertk	high autofluorescence, SiglecF, and CD11c	(25, 26, 30–32)
IM		CD11b and CX3CR1	(22, 24–26, 30, 32)
In human			
AM	HLA-DR, CD11b	CD169, MARCO	(27–29)
IM		CD36	(28)

**Table 2:** Properties of AM and IM in steady state.

	AM	IM	Reference
Origin	fetal liver monocytes	BMDM and embryonic progenitor cells	(24, 33, 34)
Replenishment	self-renew	circulating monocytes, self-renew	(24, 27, 31, 35)
Location	lumen of the alveoli	parenchyma near bronchi, nerves, and blood vessels	(24, 27, 31, 32, 36, 37)
Morphology	larger size, more pseudopodia	smaller size, irregularly shaped nuclei	(26, 30, 38)
Characteristic	long-lived cells, slower turnover rate, higher adhesion ability	short-lived cells, high turnover rate	(39)
Function	higher phagocytic capacity	immunoregulatory, higher antigen presenting capacity	(24, 26, 30, 38, 40–45)

## Under-appreciated interstitial macrophages in lung fibrosis

Much is known about AMs due to their abundance at steady-state, and most of the studies on macrophages during lung fibrosis have focused on AMs, partially due to IMs being less understood, and there are currently a limited number of surface markers to characterize IMs in steady-state and in fibrotic lungs. However, recent studies using a single-cell genomics approach to study tissue-resident immune cells of the lungs have revealed that IMs may be underappreciated during lung fibrosis.

In lung fibrosis, AMs alter collagen synthesis and stimulate fibroblasts through the secretion of inflammatory mediators, such as CCL18, MFG-E8, FSP-1, and MMPs (48–52). Previous studies suggest that monocyte-derived AMs may be involved in fibrosis formation through their interactions with fibroblasts (53). AMs originating from hematopoietic stem cells (HSC) probably play a crucial role in the resolution of inflammation because the lung microenvironment involved in regulating macrophage epigenetic function is altered during differentiation from monocytes (35, 36, 54–57). However, it isn’t clear if IMs could have similar or opposing roles to AMs during lung fibrosis. IMs have been studied significantly less than other immune cells present in fibrotic lungs (20, 22, 26, 57). In recent years, IMs have been studied mainly in responses to different environmental stimuli (such as house dust mite antigen) (26, 40), lung injury (such as acute lung injury and hypoxia) (26, 58, 59), and infectious conditions (such as influenza and *Streptococcus pneumoniae*) (26). However, studies of IM function in pulmonary fibrosis are very limited. Previous studies have reported that IMs may consist of subgroups with different phenotypes, and a significant increase in IMs has been observed in a bleomycin (BLM)-induced fibrosis model (30, 47), which suggests that IMs may have a crucial role in pulmonary fibrosis. Therefore, further study is required to understand the role of IMs in the pathogenesis of pulmonary fibrosis.

## The origin and maintenance of interstitial macrophages

Macrophages in the lungs have diverse origins *in vivo* that have been delineated by multiple genetic murine models, including parabiotic mice, fate mapping tools, and bone marrow chimeras. AMs are derived mainly from fetal liver monocytes instead of the other sources, such as yolk-sac-derived progenitors or bone marrow-derived monocytes (BMDM) (33). Post-natal development of AMs occurs a week after birth when they display a CD11b^med^ Ly6C^med^ CD11c^hi^ SiglecF^hi^ phenotype, after that AMs can undergo homeostatic self-renewal (35, 60). Interestingly, under certain pathological conditions, such as in fibrotic lungs, monocyte-derived macrophages (MDMs) can replenish AMs (36, 61). However, the origin and developmental input of IMs is less well defined. Like AMs, pulmonary IMs are considered a heterogeneous population that includes macrophages from different origins (22, 34).

Functionally, IMs might be important for the homeostasis of the bronchi, nerves, and blood vessels in the lungs, termed as peri-bronchial, peri-nerval and peri-vascular IMs, respectively (24, 31, 32, 36). IMs can originate from BMDM and embryonic progenitor cells (24, 34). MDMs may contribute to two subsets of IMs, Lyve1^lo^ MHCII^hi^ IMs and Lyve1^hi^ MHCII^lo^ IMs (24). In addition, fate-mapping experiments have shown that macrophages derived from yolk-sac progenitors can persist in the lungs. Still, they can be replaced by blood monocyte-derived IMs after birth (34). In previous studies, an *in vivo* fate-mapping system was used to illustrate the source of replenishment for IMs. This fate-mapping model labeled cells derived from fetal monocytes using Cre recombinase expression under the control of a S100a4 promoter, which turned on an EYFP reporter at the murine Rosa26 locus (62). Interestingly, only IMs were labeled by the fluorescent reporter in adult mice, indicating that monocytes give rise to IMs (24). Radiation chimera experiments with CD45.1 WT and CD45.2 CCR2 KO bone marrow progenitors suggest that the two populations (Lyve^lo^ MHCII^hi^ and Lyve^hi^ MHCII^lo^) of IMs are equivalently replenished by circulating monocytes, while AMs are not (24). Using an adoptive transfer mouse model (63), Ly6C^hi^ monocytes from peripheral blood were adoptively transferred into a macrophage-depleted recipient, which confirmed that Ly6C^hi^ monocytes replaced IMs in the lungs (24). Parabiotic mouse models have also demonstrated that circulating monocytes can maintain the IM population (34). Meanwhile, CD206^+^ and CD206^−^ IMs were demonstrated to self-renew in a short period of time, and CD206^+^ IMs are more prone to self-maintenance (31). IMs can be replaced by monocytes and through self-renewal in steady-state, however, their origins during an inflammatory response are unclear.

## Heterogeneity of interstitial macrophages

The heterogeneity of IMs remained less characterized, partly due to technological limitations that prevent accurate isolation and discrimination of IMs from monocytes and dendritic cells (64). However, with the increased use of scRNA-seq technology in recent years, the dissection of immune cells present in tissues, such as the lungs, has experienced an unprecedented improvement in precision and throughput (65). Furthermore, this technique has provided novel insights into the identification and classification of heterogeneous macrophages in the lungs, particularly during disease states.

The classification of IM subsets has greatly evolved with these new experimental tools. An earlier classification of three IM subsets was based on bulk RNA-sequencing from sorted cells, isolated by surface markers such as CD11c^lo^ MHCII^lo^, CD11c^lo^ MHCII^hi^, and CD11c^+^ MHCII^hi^ (25). All IMs were found to share MERTK, CD64, CD11b, and CX3CR1 expression, and they were located in the bronchial interstitium but not the alveolar interstitium (25). More recently, two independent subpopulations of IMs were defined by scRNA-seq in mice. These two groups of cells, namely Lyve1^lo^ MHCII^hi^ CX3CR1^hi^ (Lyve1^lo^ MHCII^hi^) IMs and Lyve1^hi^ MHCII^lo^CX3CR1^lo^ (Lyve1^hi^ MHCII^lo^) IMs, are located near nerves and blood vessels, respectively (24). Four subpopulations of macrophages were found in sorted non-autofluorescent CD45^+^ SSC^lo^ F4/80^+^ CD11c^−^ Ly6C^lo^ CD64^+^ cells from the lungs using scRNA-seq (31). One subpopulation was AMs and two other subpopulations were found with typical IM marker expression, including Cx3cr1, Mafb, Cd14, and Cd74, and were differentiated as either CD206^+^ IMs and CD206^-^ IMs. The final subset was previously undefined and characterized as CD64^+^ CD16.2^+^ monocytes (31). While CD206^+^ IMs are mainly located in the bronchial interstitium, CD64^+^ CD16.2^+^ monocytes and CD206^-^ IMs were present in the alveolar interstitium (31). The author’s analysis further concluded that CD206^-^ IMs overlapped with Lyve1^lo^ MHCII^hi^ nerves and the CD11c^+^ MHCII^hi^ group, and CD206^+^ IMs overlapped with the Lyve^hi^ MHCII^lo^ blood vessel group, CD11c^lo^ MHCII^lo^, and CD11c^lo^ MHCII^hi^ group of cells (31). More recently, IMs were characterized as Lyve1^hi^ CD206^hi^ IMs and CX3CR1^hi^ MHCII^hi^ IMs based on the above research (36). CD64^+^ CD16.2^+^ monocytes are a group of intermediate cells that are linked to monocytes and IMs. RNA velocity, an indicator of dynamic changes in transcripts that predict future cell state changes, showed a tendency for Ly6C^lo^ to transfer to CD64^+^ CD16.2^+^ monocytes and then to CD206^-^ IMs (31).

## Involvement of interstitial macrophages in lung fibrosis

Recent research has highlighted that IMs may play an important role in initiating pulmonary fibrosis. In both human samples and animal models, IMs have been found to accumulate in fibrotic lungs (30, 47, 68, 70). In addition, recent scRNA-seq experiments in murine lung fibrosis models suggest that IMs can respond to stimuli under fibrotic conditions, such as bleomycin (46) and radiation (66). As discussed above, it has also been shown that IMs may represent more than a single subset of cells.

scRNA-seq analysis has been used to classify macrophages in mice with a bleomycin-induced lung fibrosis model (46). Macrophages were divided into three main populations; AMs, IMs, and intermediate populations, and the intermediate cluster was subsequently defined as SiglecF^+^ CD11c^+^ MHCII^hi^. The intermediate and IM populations were mainly enriched in the bleomycin group and expressed markers characteristic for monocytes (46). In the absence of Lyve1^hi^ MHCII^lo^ IMs, the infiltration of inflammatory cells, such as monocytes, was elevated in the lungs of mice from a bleomycin-induced pulmonary fibrosis model, confirming that Lyve1^hi^ MHCII^lo^ IMs attenuate lung fibrosis (24). In the radiation-induced fibrosis (RIF) mouse model, IMs were first demonstrated to have a different role than AMs: Depletion of IMs with colony-stimulating factor receptor-1 (Csf1r) neutralizing antibody revealed a significant decrease in CD206^+^ IMs and alleviation of fibrosis symptoms, suggesting that IMs may play a pro-fibrotic role in the RIF processes through the CSF1/CSF1R pathway (66). More recently, macrophages sorted based on MERTK^+^ CD64^+^ expression were found to have two clusters of AMs and three clusters of IMs (32). Among the IMs, a subset of cells highly overlapped with CD169^+^ CD11c^-^ nerve and airway-associated macrophages (NAMs), and their development and survival depended on the CSF1-CSF1R signaling pathway (32). PR8 influenza virus and TLR3 ligand poly(I:C) validated that NAMs play a major role in immune regulation and tissue homeostasis under inflammatory conditions (32). NAMs are related to the immune response and highly express CD206 and may largely overlap with CX3CR1^hi^ MHCII^hi^ IMs (32). Therefore, this NAM subset may play a role in lung fibrosis and deserves more functional studies in fibrotic models.

However, there are some controversial findings relating to the role of AMs. One such study used an asbestos-induced lung fibrosis murine model to identify macrophages based on typical macrophage-associated genes (including CD68, Mrc1, Lyz2, Adgre1 and Axl) using scRNA-seq. Then the cluster expressing Mrc1 was re-clustered in which CD68 was regarded as a pan-macrophage marker, Car4 was a mature tissue-resident alveolar macrophage (AM1 and AM2) marker, Mmp12 was a monocyte-derived alveolar macrophage (AM3 marker), and tissue-resident IMs were characterized by Cx3cr1 expression, then divided into perivascular IMs (mainly by Lyve1 expression) and peribronchial IMs (mainly by Ccr2 expression). AM2 and AM3 were predominantly expressed in the asbestos-treated group, and AM3 was enriched in more pulmonary fibrosis-related genes (67). Next, the authors analyzed a previously published bleomycin-induced pulmonary fibrosis model dataset and found that AM3 was predominantly present in the BLM-induced fibrosis group (67).

In IPF patients, scRNA-seq technology has also been used to investigate the heterogeneity and function of macrophages by comparing cells taken from fibrotic lower lobes, upper lobes, and normal lungs (68). Macrophages expressing CD163 and AIF1 were identified in three subsets: a subset that highly expressed FABP4 and INHBA, defined as AMs predominantly present in alveolar lavage fluid, a subset defined as IMs (seldom observed in BAL) predominantly expressing SPP1 and MERTK, and a subset associated with monocytes with high FCN1 expression. In addition, the SPP1 and MERTK IMs had increased prevalence in pulmonary fibrosis patient samples (68). In another study, macrophage datasets from IPF patients and lung transplant donors were combined for analysis, and macrophages were divided into four populations, two mainly from healthy samples present tissue-resident AMs in homeostasis and two from pulmonary fibrosis samples. In addition, CHI3L1, MARCKS, IL1RN, PLA2G7, MMP9, and SPP1 genes were only expressed in patients’ fibrotic lesions (69). Another study used scRNA-seq to assess data from IPF patients, COPD patients, and healthy controls. Macrophages and monocytes were defined as four clusters, whereby AMs were defined as FABP4^hi^ and C1QB^+^ cells, IMs were defined as ITGAM^hi^ cells, classical monocytes were defined as CD14^+^ cells, and non-classical monocytes were defined as CD16^+^ CD14^-^ cells (70). IMs from IPF samples expanded, but such expansion was not found for IMs from healthy and COPD samples. Moreover, CD84^++^ CD36^++^ macrophage subpopulations in IPF samples were increased compared to healthy and COPD samples (70).

Therefore, analysis of lung macrophages subsets by scRNA-seq allowed for identifying both AM and IM subpopulations. This refined classification is still diverse among different studies, which may be due to differences in sample preparation, data analyses, murine model development, and the use of other species. 1) Lung samples are often prepared using digestive enzymes, such as collagenase and DNase, however incomplete and inadequate digestion can result in low yields of macrophages and DCs (71). 2) Specifying cell populations present in samples is dependent on the use of various marker genes. As such, researchers may select different genes to define specific macrophage subpopulations, however the use of different combinations of marker genes could make subpopulations be reported differently across various studies. The marker gene selected may be related to the disease, for example the gene Mmp12 was considered a subset macrophage marker (AM3) and AM3 was predominantly present in the BLM-induced fibrosis group (67), while Mmp12 is reported to participate in lung fibrosis (72, 73). 3) Different mouse models of lung fibrosis could also lead to discrepancies in reporting. Different stimuli, such as pathogen-associated molecular patterns (PAMPs), including CpG DNA, LPS, Poly (I:C), and FLA-BS, are known to lead to variations in macrophage responses. These PAMPs act as ligands for different Toll-like receptors (TLRs). Only CpG DNA (TLR9 ligand) has been shown to have the most potential in altering macrophages in the IM population (26). Different drug-delivery methods when inducing disease models can cause IMs and AMs responses to vary. For example, intranasal LPS delivery induces the AM population to strongly respond, while intraperitoneal injection of LPS induced stronger IMs responses (41). 4) The gene expression profile of different species may result in discrepancies between murine and human reports involving scRNA. The AMs and IMs populations of both murine and human were sorted out for scRNA sequencing, and the gene expression profile revealed that some genes (SERPING1, MME, IL17RB, and C1Q) were more expressed in human AMs, while some genes (SPP1, MMP8, and CARD11) were more inclined to be expressed in murine macrophages. Besides, the IMs of humans and mice are not strictly corresponding (28). A strain-specific bias can also be seen when comparing the transcriptomes of different mouse strains (41). A more detailed description of different strategies in identifying lung macrophages and their association with lung fibrosis is summarized in **Table 3**. However, it is certain that in both steady-state and fibrotic lungs, IMs consist of more than one subpopulation, and IM subsets respond to fibrotic stimuli. The heterogeneity of IMs in lung fibrosis deserves additional in-depth fate-mapping studies and functional studies to understand how such subsets contribute to disease pathology.

**Table 3:** scRNA sequencing for lung macrophage clustering in lung fibrosis and steady-state.

Analyzed samples	Identification markers	Subset of macrophage	Signature genes	Characteristics	Involvement in lung fibrosis	Reference
Mouse lung	ND	AM	ND	correspond with SiglecF^+^ CD11c^+^ MHCII^lo^ cells	present in steady-state and bleomycin-induced lung fibrosis model	(46)
		AM-IM-intermediate	Cx3cr1, Ccr2, Mafb, MHCII	correspond with SiglecF^+^ CD11c^+^ MHCII^hi^ cells, transition to AM during bleomycin-induced pulmonary fibrosis	mainly present in bleomycin-induced lung fibrosis model, localized to the fibrotic niche	
		IM	Cx3cr1, Ccr2, Mafb, MHCII	ND	mainly present in bleomycin-induced lung fibrosis model	
Mouse lung	Cd68, Mrc1, Lyz2, Adgre1, Axl as macrophage; Siglecf, Marco, Il18 as AM, Cx3cr1 as IM	AM1	Car4, Ear1, Fabp1	maintain homeostasis	present in TiO_2_ and asbestos-induced lung fibrosis model	(67)
		AM2	Car4, Ctsk, Chil3, S100a1, Wfdc21	inflammatory response, cytokine secretion, and matrix metalloproteinase activation	mainly present in asbestos-induced lung fibrosis model	
		AM3	Mmp12, Itgam, Cd36 and Gpnmb, Litaf, Jund, Bhlhe40, Bhlhe41, Klf9, Atf3, Atf4	immature alveolar macrophage phenotype, macrophage development and maintenance, unfolded protein response.	mainly present in asbestos-induced lung fibrosis model, enriched for pro-fibrotic genes	
		IM1	Lyve1	surrounding blood vessel	ND	
		IM2	Ccr2	surrounding bronchia	ND	
Human lung	AIF1, CD163	AM(FABP4^hi^)	FABP4, INHBA	lipid metabolic function	higher rates of proliferation in IPF lungs, decreased in lower lobes IPF patient sample	(68)
		IM (SPP1^hi^)	SPP1, MERTK, LGMN, SIGLEC10	stress response, causally related to fibroblasts	higher rates of proliferation in IPF lungs, increased in lower lobes IPF patient	
		monocyte/macrophage (FCN1^hi^)	FCN1, CD14, IL1B, INSIG1, OSM, IL1R2, THBS1	immune response function, smaller cells	lower rates of proliferation in IPF lungs, decreased in lower lobes IPF patient sample	
Human lung	ND	AM	FABP4, C1QB	ND	AMs occupy 49% of IPF-expanded macrophages, the terminal phenotype in IPF	(70)
		IM	ITGAM	ND	IMs occupy 51% of IPF-expanded macrophages, the intermediate cell-state in IPF	
Sorted murine MerTK^+^ CD64^+^ from lungs	ND	IM1	MHCII genes (H2-Aa, H2-Ab1, H2-DMb1, H2-Eb1), Cx3cr1	correspond with Lyve1^lo^ MHCII^hi^ cells, immune response activation and antigen presentation, surrounding nerves	steady-state	(24)
		IM2	Tfgb2, Plaur	correspond with Lyve1^hi^ MHCII^lo^ cells, wound healing, surrounding blood vessels, larger cells	steady-state	
Sorted murine * F4/80^+^ CD11c^-^ Ly6C^lo^ CD64^+^ from lungs	Cx3cr1, Mafb, Cd14, Cd74	IM1(CD206^-^)	MHCII genes (H2-Eb1, H2-Ab1, Cd74)	processing and presentation of antigen, regulate T cell response	steady-state	(31)
		IM2(CD206^+^)	Mrc1, Cd163, Lyve1, Folr2	regulation of leukocyte chemotaxis, wounding and endocytosis, larger cells	steady-state	
		monocyte/IM3(CD64^+^ CD16.2^+^)	Ace, Fcgr4	cell adhesion, integrin-mediated signaling pathway, regulation of cytoskeleton structure and migration	steady-state	
Sorted murine MerTK+ CD64+ from lungs	ND	AM1	ND	ND	steady-state	(32)
		AM2	ND	ND	steady-state	
		IM1(CD169^+^ CD11c^-^, NAMs)	C1qa, C1qb, C1qc, MHCII genes (H2-Eb1, H2-Ab1, H2-Aa), Mgl2, Cd83, Apoe, Pf4, Tmem176a	immunoregulatory, elongated cells, surrounding airways, nerve-associated	steady-state	
		IM2(CD11c^+^ CD169^−^)	Icam2, Ly6a, Lyve1	ND	steady-state	

Characteristics are mainly defined by transcriptomic analysis.

AM, alveolar macrophage; IM, interstitial macrophage; *, CD45^+^ non-autofluorescent SSC^lo^; ND, not described.

## Functions of interstitial macrophages in pulmonary fibrosis

**Figure 1:**
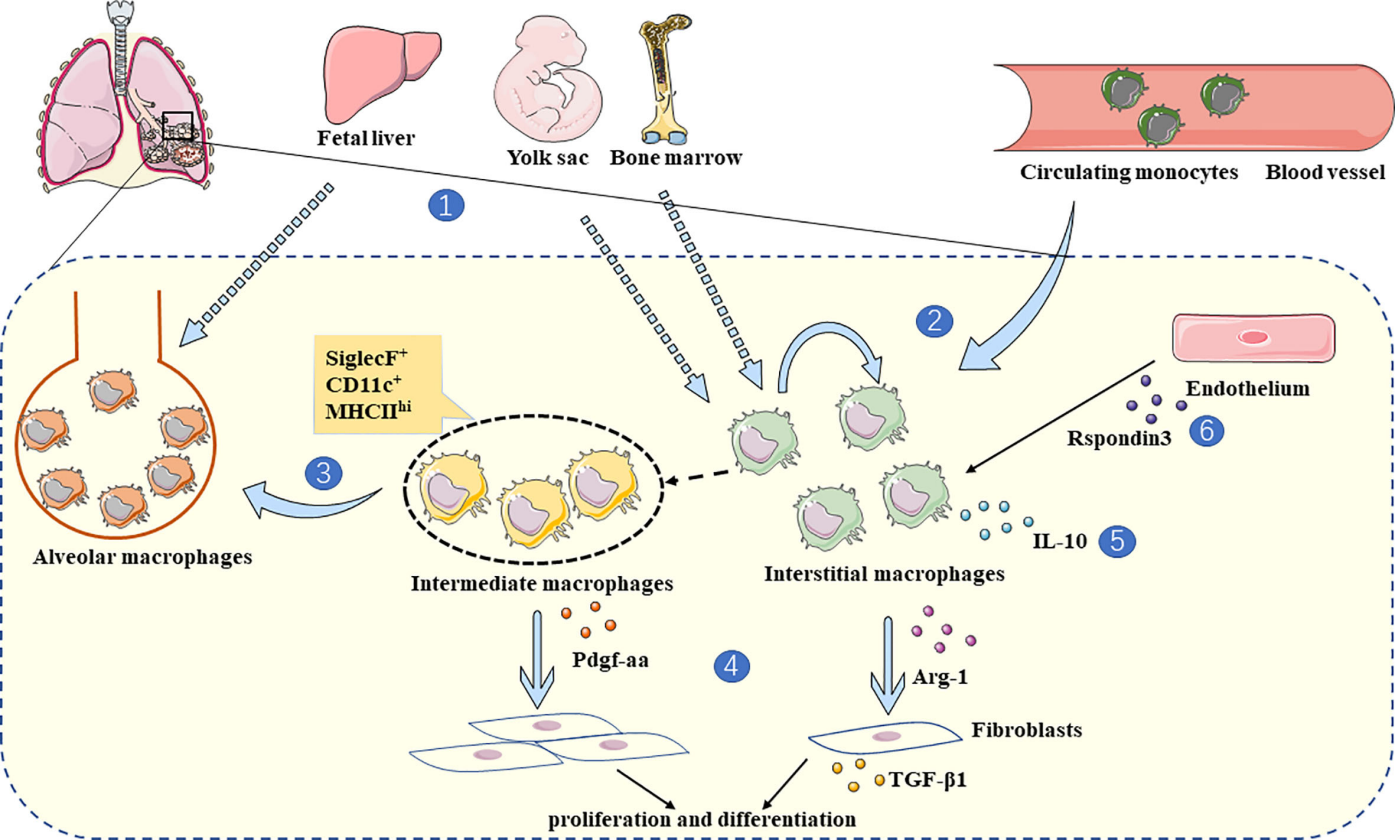
The role of IMs in lung fibrosis. **1.** IMs are derived from BMDM and yolk-sac (24, 34), while AMs are mainly derived from fetal liver (33). **2.** IMs are replenished by circulating monocytes and self-renewal (24, 31). **3.** Due to computational trajectory tracking analysis, monocytes tend to transform into IMs during steady-state (31), and intermediate macrophages with monocytic origin (SiglecF^+^ CD11c^+^ MHCII^hi^) convert to AMs in fibrotic lungs (46). **4.** In a state of lung fibrosis, IMs produce Arg-1 and interact with fibroblasts directly, which stimulates fibroblasts to produce TGF-β1 (66). In addition, intermediate macrophages (SiglecF^+^ CD11c^+^ MHCII^hi^) secrete Pdgf-aa, promoting fibroblast proliferation and differentiation (46). **5.** The major IL-10 producing IMs (lyve^hi^ MHCII^lo^) alleviate lung fibrosis (24). **6.** At the inflammatory stage of bleomycin induced lung fibrosis, Rspondin3 secreted by endothelium can promote the IMs expansion (74).

IMs contribute to pulmonary fibrosis’s pathological development, largely dependent on secreted cytokines, inflammatory mediators, and interactions with other cells in the lung parenchyma (**Figure 1**).

In steady-state, most IMs and a proportion of inflammatory monocytes produce IL-10 among pulmonary myeloid cells (40). IMs produce more IL-10 when stimulated by inflammatory triggers, such as unmethylated CpG DNA. It has been confirmed that IMs, but not AMs, play critical roles in the presence of CpG DNA stimulation-induced IL-10 secretion (26). After treatment of IL10-GFP mice with poly(I:C), it was revealed that IL-10 was mainly produced by a specific subset of IMs, NAMs (32). Numerous studies have shown that IL-10 is closely associated with fibrosis in lung and other tissues through macrophages (75–79). Lyve1^hi^ MHCII^lo^ IMs secrete more IL-10 during steady-state, and in the absence of these IMs, the symptoms of pulmonary fibrosis were more severely manifested as measured by weight loss, collagen deposition, and immune cell infiltration (24). Such discoveries indicate that IMs participate in lung fibrosis through IL-10.

Crosstalk between IMs and other cell populations has been observed during the disease progression of pulmonary fibrosis. For example, endothelial cells can regulate the expansion of IMs during the inflammatory phase in a bleomycin-induced mouse model through Rspondin3 (74). *In vitro*, IMs sorted from a RIF model were co-cultured with fibroblasts which resulted in TGF-β1 secretion, suggesting differentiation to myofibroblasts, however AMs sorted from the same model could not promote this phenotype (66). TGF-β1 is necessary for the promotion of fibroblast differentiation, and it regulates fibrosis through canonical signaling pathway TGF-β/Smad2/3 or non-canonical signaling pathways, such as ALK1/Smad1/5, ERK, JNK/p38, cAbl, PI3K/AKT, ROCK, and JAK/STAT3 (80, 81). M2-polarized macrophages are associated with TGF-β expression and are involved in lung fibrosis *via* MMP-28 and MBD2 (82, 83). Macrophage-produced TGF-β could promote lung fibrosis by inducing fibroblast differentiation (84, 85). In addition, co-culture of SiglecF^+^CD11c^+^MHCII^hi^ intermediate macrophages from a BLM-induced mouse model increased fibroblast proliferation through the secretion of Pdgf-aa (46). These studies suggest that IMs can exacerbate fibrosis by interacting with fibroblasts directly and influenced by endothelial cells.

During a RIF model, the expression of Arg1 in IMs was increased by 400-fold after radiation compared to the baseline and 40-fold compared to stimulated AMs. IL-13/IL-4-activated IMs could also increase extracellular matrix and α-SMA expression in fibroblasts (66). IL-4 and IL-13 are cytokines that promote the polarization of macrophages toward an M2-phenotype and Arg1 is a typical marker for M2 macrophages (86). Moreover, plenty of evidence suggests that M2 macrophages produce the anti-inflammatory cytokine IL-10 (75, 87, 88). These studies suggest that the roles of IMs in fibrosis may be closely related to their M2 polarization.

Intermediate macrophages (SiglecF^+^ CD11c^+^ MHCII^hi^) can convert to AMs during BLM-induced lung fibrosis progression (46). Differentiation of IMs into AMs during the bleomycin-induced fibrosis phase has also been observed (30, 47). Recent study found that resident AMs were replaced by a population of newly arrived monocyte-derived IMs during bleomycin-induced lung fibrosis. The transition macrophage is characterized as SiglecF^+^ CD11b^+^ and CD206^hi^ (85). Research on IPF patients also found that IMs could differentiate into AMs, and M2 macrophages were present in the early stages of fibrosis (70). Moreover, the features of macrophages in IPF patients could change over time, associated with early-phase expression of SPP1 and LIPA, followed by MMP9, SPARC, PALLD, CTSK, and GPC4, and at the terminal stage CSF1 expression can be detected (89). Of note, CD163/LGMN macrophages have been suggested to play a pro-fibrotic role in the lungs of COVID-19 patients, where there is a tendency for conversion from monocytes to pro-fibrotic macrophages and eventually AMs (90). To conclude, the phenotype of pulmonary macrophages has differential trajectories during lung fibrosis and IMs may act as an intermediate cell type linking monocytes and AMs. Accumulating evidence suggests that macrophages are dynamically regulated by epigenetic mechanisms, such as DNA methylation, histone modification, and chromatin structure. The lung microenvironment also provides stimulation that impacts macrophages (91, 92). The mechanisms involved in driving these macrophage responses still need to be further explored.

## Conclusion

Advanced technologies have facilitated a deeper insight into macrophages’ origins, heterogeneity, and functions in health and disease. However, IMs have historically been poorly understood due to limited molecular markers and in-depth analysis of their functions at a single-cell level. The application of scRNA-seq technology in identifying lung macrophage subpopulations under steady-state and in pulmonary fibrosis models has greatly advanced our knowledge in this field. These studies have greatly refined the description of macrophage subpopulations in the lung and provided a wealth of publically available data for the functional exploration of these cells by the scientific community. However, there are still some limitations, including differences in the depth of sequencing and sample acquisition strategies between studies, which may prevent accurate interpretation of the data from different sources.

As the classification and function of IMs in lung fibrosis are gradually uncovered, their polarization status during pulmonary fibrotic disease needs to be more rigorously studied. Although phenotypic conversion between different subsets of lung macrophages has been observed, the functional validation and underlying mechanisms require further exploration. The crosstalk between IMs and other cell types requires further characterization. In addition, the classification of macrophages may vary under differing stages of fibrosis. Using a lineage-tracing system in IMs will probably be an important aspect of future studies assessing the dynamics of this subpopulation of macrophages during lung fibrosis.

## Author contributions

YG performed the scientific literature search and wrote the manuscript, YML, TL, RM, and BHY designed the review structure and revised the review. All authors contributed to the article and approved the submitted version.

## Funding

This study was supported by Education Department of Henan Province (No. 21IRTSTHN030, No. 222300420015), and Science and Technology Department of Henan Province (No. GZS2021002).

## Acknowledgments

We would like to thank Zhuangzhuang Liu and Lichen Zhang for their assistance in preparing the manuscript. **Figure 1** used cartoon elements from Servier Medical Art (https://smart.servier.com/).

## Conflict of interest

The authors declare that the research was conducted in the absence of any commercial or financial relationships that could be construed as a potential conflict of interest.

## Publisher’s note

All claims expressed in this article are solely those of the authors and do not necessarily represent those of their affiliated organizations, or those of the publisher, the editors and the reviewers. Any product that may be evaluated in this article, or claim that may be made by its manufacturer, is not guaranteed or endorsed by the publisher.
